# Supplementary material for: Assessing the Suitability of CHA2DS2-VASc for Predicting Adverse Limb Events and Cardiovascular Outcomes in Peripheral Artery Disease Patients with Percutaneous Transluminal Angioplasty: A Retrospective Cohort Study
Source: Biomedicines. 2024 Jun 20;12(6):1374. doi: 10.3390/biomedicines12061374 (PMC11202305; doi:10.3390/biomedicines12061374)
Supplement: Supplementary file 1 [file biomedicines-12-01374-s001.zip › Supplementary Materials.pdf]

Supplementary Material

Assessing the Suitability of CHA_2DS_2-VASc for Predicting Adverse Limb Events and Cardiovascular Outcomes in Peripheral Artery Disease Patients with Percutaneous Transluminal Angioplasty: A Retrospective Cohort Study

Yu-Tsung Cheng [1], Fu-Lan Chang [2], Po-Hsien Li [3], Wen-Chien Lu [4] and Chien-Shan Chiu [5,6,7,*]

[1] Cardiovascular Center, Taichung Veterans General Hospital, 1650 Section 4 Taiwan Boulevard, Xitun District, Taichung 40705, Taiwan
[2] Department of Nursing, Taichung Veterans General Hospital, 1650 Section 4 Taiwan Boulevard, Xitun District, Taichung 40705, Taiwan
[3] Department of Food and Nutrition, Providence University, 200 Section 7, Taiwan Boulevard, Shalu District, Taichung City 43301, Taiwan
[4] Department of Food and Beverage Management, Chung-Jen Junior College of Nursing, Health Sciences and Management, 217, Hung-Mao-Pi, Chiayi City 60077, Taiwan
[5] Department of Dermatology, Taichung Veterans General Hospital, 1650 Section 4 Taiwan Boulevard, Xitun District, Taichung 40705, Taiwan
[6] Department of Post-Baccalaureate Medicine, College of Medicine, National Chung Hsing University, 145 Xingda Road, South District, Taichung 402, Taiwan
[7] College of Biotechnology and Bioresources, Da-Yeh University, 168, University Road, Dacun, Changhua 51591, Taiwan
* Correspondence: chienshan@vghtc.gov.tw

I. Supplementary Materials

Clinical parameter description

Other diagnostic, clinical, and environmental factors were available for all PAD patients, defined as follows: smoking history classified as ever- and never-smokers, where ever-smokers were smokers who quit or were current smokers; ≧10 cigarettes a day for at least 1 year without an attempt to quit; with or without hyperlipidemia (HPL), based on a predefined low-density lipoprotein (LDL) cholesterol threshold set by the National Cholesterol Education program (<70 mg/dl for PAD patients; and <55 mg/dl for coronary artery disease (CAD) patients) [1], whether or not on lipid-lowering medications, and/or newly diagnosed patients at the time of hospitalization; history of CAD; history of coronary artery bypass graft (CABG), history of percutaneous cardiac intervention (PCI), history of old myocardial infarction (MI), history of chronic obstructive pulmonary disease (COPD), history of chronic kidney disease (CKD) with a creatinine clearance test threshold value of CCr.: <60ml/min, history of hemodialysis (HD) or peritoneal dialysis (PD), history of atrial fibrillation (AF), history of autoimmune disease, information on blood marker levels, such as glycated hemoglobin (HbA1C), cholesterol, LDL, high-density lipoprotein (HDL) cholesterol, triglyceride (TG), and glucose. Information on medications were also included, such as aspirin (ASA), clopidgrel, cilostazol, pentoxyphilline, warfarin or direct oral anticoagulant (DOAC), angiotensin-converting enzyme inhibitors (ACEI) or angiotensin receptor blockers (ARB), statin, betablocker, calcium channel blockers (CCB), and insulin.

The mean age of patients with MACE was higher than that of patients without MACE, while the mean age of MALE patients was slightly lower than that of those without MALE. Furthermore, patients demonstrating MACE had a significantly higher proportion of CHF, HTN, and DM when compared to patients without MACE, but the trend was reversed in cases with a MALE event. Hyperlipidemia was observed in a significantly higher number of patients with MACE (P = 0.05) outcomes when compared against those with no event. Smoking (ever) was observed to be correlated in direct proportion to MACE occurrence (46.93% (MACE); 37.83% (no MACE)), but was inversely and proportionally correlated with MALE. Occurrence of other clinical characteristics, such as CAD, CABG, PCI, Old MI, COPD, CKD, and AF was significantly greater in PAD patients with MACE (P<0.0001) in comparison to those without MACE, but the occurrence was low in patients with MALE as compared with their non-MALE counterparts. Autoimmune diseases were less prevalent or absent in patients with MALE and MACE, respectively. Mean ± standard deviation (SD) of all diagnostic blood markers are provided in Table 1; no

blood markers were significantly different between MACE and non-MACE, and except for HbA1C (P=0.001) and glucose levels (P=0.02), none showed a significant difference between MALE and non-MALE. Medication usage was also not significantly different among all of the groups with and without events.

Model evaluation

A 10-fold cross-validation process was implemented to confirm the predictive performance of the MCR score and to confirm its reproducibility [15]. Also, the MCR-based regression models' predictions were compared with traditional models that used clinical parameters directly instead of the cumulative score. This was done to determine whether using MCR as the predictor demonstrated any improvement over the traditional methods. **Table 4** lists the average and the standard deviations of the c-indices over all 10CVs for all three regression models for MALE, MACE, and MALE+MACE. The results provide evidence that for a given patient pair, MCR can effectively discriminate the occurrence of MACE events, which indicates it to be a good predictor. However, the discrimination power of MCR for MALE and MALE+MACE were not good enough.

Next, calibration analyses were conducted using 10CV for each of the 5 years for all models. The difference between the proportions for the predicted and observed events were averaged and plotted for MCR models in comparison to the traditional model. For MACE, the differences of the proportions between observed and predicted for both MCR and traditional models were ≤5% for the first 2 years, and for the years 3-5 were restricted to <10%, while for both MACE and MALE, the average of differences was quite high (33-49%), except for year 1 (<5%). **Figure 4**, and **Table S4–S9** showcases detailed calibration results.

Further evaluation of MCR was conducted using ROC analysis for predicting MALE, MACE and MALE+MACE at time points of 12months, 24 months, 36 months and 48 months, respectively (**Figure 5**). For outcome MACE, the univariate model and the multivariate adjusted model demonstrated a maximum AUC of ~0.66 and ~0.85 for time points 48 months and 12 months, respectively **(Figure 5B,E)**, while for the both outcomes MALE and MALE+MACE the univariate model demonstrated AUCs of <0.6 (**Figure 5A,C**) for all time points whereas the maximum AUC for multivariate models was ~0.65 for time points >36 months (**Figure 5D,F**). Based on all of our findings, we can fairly say that CHA_2DS_2-VASc can be claimed as a good predictor of MACE but doesn't qualify as a good predictor of MALE and MALE+MACE for patients with PAD.

1 Supplementary Figures and Tables

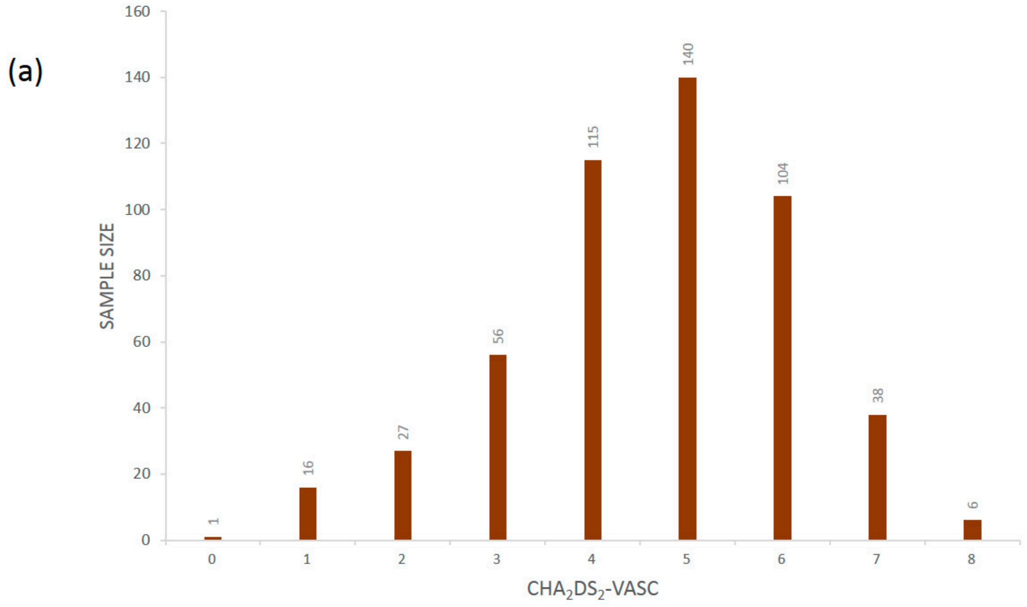

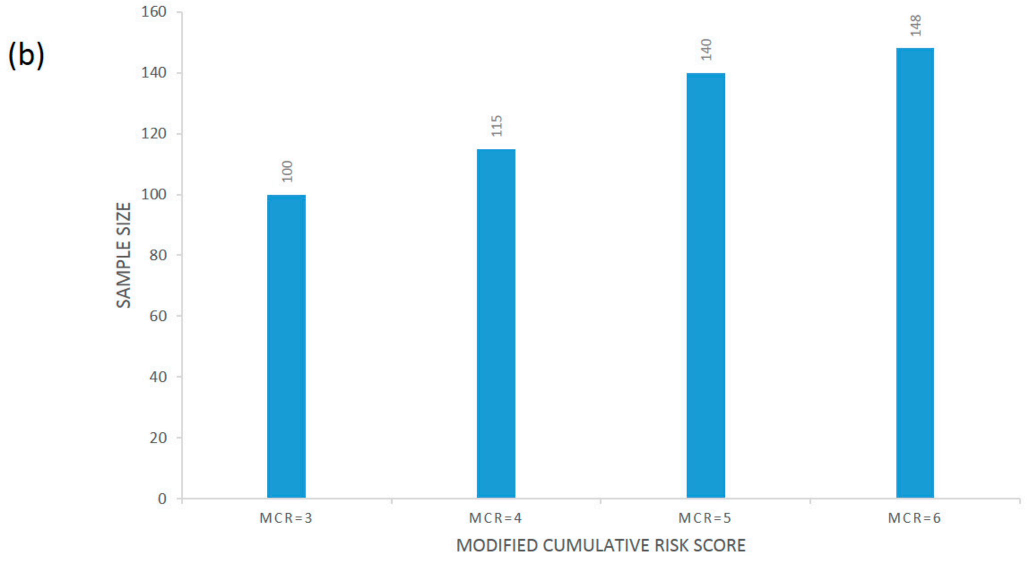

Figure S1. The distribution of PAD samples based on (a) CHA_2DS_2-VASc and (b) MCR scores

Table S1. Description of Rutherford classifications

Grade	Category	Clinical description	Objective criteria
0	0	Asymptomatic – no hemodynamically significant occlusive disease	Normal treadmill test and ABI ≥ 0.9
	1	Mild claudication	Treadmill test completed. AP after exercise <50 mmHg
I	2	Moderate claudication	Between categories 1 and 3
	3	Severe claudication	Cannot complete treadmill test and Ankle pressure (AP) after exercise <50 mmHg
II	4	Ischemic rest pain	Resting Ankle pressure (AP) <40 mmHg, flat or barely pulsatile ankle or metatarsal PVR, TP <30 mmHg
III	5	Minor tissue loss – non-healing ulcer, focal gangrene with diffuse pedal ischemia	Resting AP <60 mmHg, ankle or metatarsal PVR flat or barely pulsatile, TP <40 mmHg
	6	Major tissue loss- extending above TM level, functional foot no longer salvageable	Similar to category 5

AP: Ankle pressure, PVR: pulse volume recording, TP: toe pressure

Table S2. Demographic and clinico-pathological characteristics for major adverse limb events (MALE) and major adverse cardiovascular events (MACE)

	MALE (n=193)	no MALE (n= 310)	P-value	MACE (n=49)	No MACE (n = 454)	P-value
MCR score						
=3	31 (16.06)	69 (22.26)		3 (6.12)	97 (21.37)	
=4	49 (25.39)	66 (21.29)		13 (26.53)	102 (22.47)	
=5	63 (32.64)	77 (24.83)		16 (32.65)	124 (27.31)	
=6	50 (25.91)	98 (31.61)	0.05*	17 (34.69)	131 (28.85)	0.0002*
Age	69.83 ± 12.44	71.35 ± 12.34	0.182	71.75 ± 10.85	70.67 ± 12.55	0.512
Sex (Male)	123 (63.73)	203 (65.48)	0.701	34 (69.39)	292 (64.31)	0.537
BMI	23.687 ± 3.665	24.14 ± 4.04	0.191	24.15 ± 3.502	23.94 ± 3.95	0.713
CHF (C)	89 (46.11)	149 (48.60)	0.7136	34 (69.39)	204 (44.93)	0.001*
HTN	165 (85.49)	271 (87.42)	0.589	47 (95.92)	389 (85.68)	0.045*
DM	158 (81.86)	218 (70.32)	0.004*	44 (89.80)	332 (73.12)	0.009*
Stroke (S)/TIA	31 (16.06)	60 (19.35)	0.405	11 (22.24)	80 (17.62)	0.434
Vascular Disease	193 (100)	310 (100)	1	49 (100)	454 (100)	1
HPL	99 (51.29)	142 (45.80)	0.235	30 (61.22)	211 (46.48)	0.05*
SMK	71 (36.78)	124 (40)	0.51	23 (46.93)	172 (37.88)	0.221
CAD	101 (52.33)	162 (52.25)	1	33 (67.49)	230 (50.66)	0.034*
CABG	18 (9.33)	35 (11.29)	0.551	10 (20.41)	43 (9.47)	0.026*
PCI	95 (49.22)	144 (46.5)	0.582	30 (61.22)	209 (46.03)	0.05
Old MI	24 (12.43)	55 (17.74)	0.13	17 (34.69)	62 (13.66)	0.0005*
COPD	7 (3.63)	14 (4.52)	0.819	5 (10.20)	16 (3.52)	0.043*
CKD	120 (62.17)	199 (64.19)	0.703	41 (83.67)	278 (61.23)	0.001*
HD/PD	73 (37.82)	108 (34.83)	0.505	22 (44.90)	159 (35.02)	0.209
Cr	3.484 ± 3.345	3.128 ± 2.846	0.236	4.237 ± 2.964	3.15 ± 3.03	0.024*
Af	41 (21.24)	79 (25.48)	0.2847	19 (38.77)	101 (22.24)	0.013*
Imd	6 (3.11)	15 (4.83)	0.492	0 (0)	21 (4.62)	0.248
HbA1C	7.682 ± 2.017	7.104 ± 1.727	0.001*	7.716 ± 1.796	7.27 ± 1.876	0.114
Cholesterol	149.63 ± 41.227	149.66 ± 38.31	0.992	143.38 ± 27.496	150.33 ± 40.46	0.115
LDL	82.87 ± 34.177	83.673 ± 32.87	0.796	76.22 ± 30.17	84.14 ± 33.61	0.089
HDL	42.238 ± 14.915	43.37 ± 15.40	0.412	43.979 ± 14.09	42.83 ± 15.34	0.591
TG	131.59 ± 84.48	130.41 ± 83.48	0.878	123.53 ± 71.24	131.66 ± 85.06	0.46
Glu	154.88 ± 74.85	139.82 ± 65.398	0.021*	149.53 ± 59.80	145.18 ± 70.50	0.636
ASA	149 (77.20)	236 (76.13)	0.829	39 (79.59)	346 (76.21)	0.723
clopidgrel	165 (85.49)	262 (84.52)	0.799	44 (89.79)	383 (84.36)	0.403
cilostazol	121 (62.69)	180 (58.06)	0.349	29 (59.18)	272 (59.91)	1
pentoxyphilline	1 (0.51)	0 (0)	0.383	0 (0)	1 (0.22)	1

direct oral anticoagulant (DOAC)	25 (12.95)	48 (15.48)	0.515	7 (14.29)	66 (14.54)	1
ACEIARB	84 (43.52)	136 (43.87)	1	17 (34.69)	203 (43.39)	0.225
statin	106 (54.92)	177 (57.09)	0.645	23 (46.93)	260 (55.51)	0.175
Betablocker	76 (39.37)	113 (36.45)	0.509	15 (30.61)	174 (37.23)	0.352
CCB	74 (38.34)	127 (40.97)	0.575	15 (30.61)	180 (37.89)	0.280
Insulin	45 (23.32)	61 (19.68)	0.368	13 (26.53)	93 (19.38)	0.356
**Rutherford classification**						
1	0 (0)	0 (0)	1	0 (0)	0 (0)	1
2	0 (0)	0 (0)	1	0 (0)	0 (0)	1
3	0 (0)	0 (0)	1	0 (0)	0 (0)	1
4	35 (18.13)	95 (30.64)	0.001*	5 (10.20)	125 (27.53)	0.009*
5	133 (68.91)	183 (59.03)	0.029*	35 (71.43)	281 (61.89)	0.215
6	25 (12.95)	32 (10.32)	0.575	9 (18.37)	48 (10.57)	0.15
**Target vessel**						
CIA	12 (6.22)	29 (9.35)	0.243	4 (8.16)	37 (8.15)	1
EIA	10 (5.18)	35 (11.29)	0.024*	5 (10.20)	40 (8.81)	0.791
CFA	10 (5.18)	17 (5.48)	1	3 (6.12)	24 (5.29)	0.739
SFA	113 (58.54)	172 (55.48)	0.518	27 (55.10)	258 (56.83)	0.879
ATA	100 (51.81)	148 (47.74)	0.409	26 (53.06)	222 (48.90)	0.653
Popliteal	50 (25.91)	57 (18.39)	0.056*	10 (20.41)	97 (21.37)	1
Peroneal artery	46 (23.83)	50 (16.13)	0.036*	13 (26.53)	83 (18.28)	0.181
Tibiofibular TP trunk	25 (12.95)	39 (12.58)	0.891	3 (6.12)	61 (13.44)	0.178
PTA	88 (45.59)	108 (34.84)	0.018*	15 (30.61)	181 (39.87)	0.221
DPA	10 (5.18)	5 (1.61)	0.029*	1 (2.04)	14 (3.08)	1
Plantar artery	15 (7.78)	8 (2.58)	0.008*	2 (4.08)	21 (4.63)	1

BMI: body mass index; CHF (C): HTN: hypertension; DM: diabetes mellitus; Stroke (S)/TIA; HPL: hyperlipidemia; SMK: smoking status; CAD: coronary artery disease; CABG: Coronary Artery Bypass Graft; PCI: Percutaneous coronary intervention; MI: myocardial infarction; COPD: chronic obstructive pulmonary disease; CKD: chronic kidney disease; HD/PD: hemodialysis/peritoneal dialysis; Cr: creatinine; Af: atrial fibrillation; Imd: immune-related disease; HbA1C: hemoglobin A1C, LDL: low-density lipoprotein; HDL: high-density lipoprotein; TG: triglyceride; Glu: glucose; ASA: Acetylsalicylic acid; ACEIARB: Angiotensin-Converting Enzyme Inhibitor (ACEI)/Angiotensin Receptor Blocker (ARB); CCB: Calcium channel blockers; CIA: common iliac artery; EIA: external iliac artery; CFA: common femoral artery; SFA: superficial femoral artery; ATA: anterior tibial artery; Tibiofibular TP (tibioperoneal) trunk; PTA: posterior tibial artery; DPA: dorsalis pedis artery.

Table S3. Demographic and clinico-pathological characteristics for outcome major adverse limb events (MALE) + major adverse cardiovascular events (MACE).

	MALE +MACE (n=224)	no MALE+MACE (n= 279)	P-value
MCR score			
=3	34 (15.17)	66 (23.66)	
=4	57 (25.45)	58 (20.79)	
=5	70 (31.25)	70 (25.09)	
=6	63 (28.13)	85 (30.47)	0.05*
Age	69.99 ± 12.26	71.39 ± 1247	0.208
Sex (Male)	147 (65.62)	179 (64.16)	0.778
BMI	23.82 ± 3.703	24.09 ± 4.06	0.443
CHF (C)	115 (51.34)	123 (44.09)	0.107
HTN	196 (87.50)	240 (86.02)	0.692
DM	185 (82.59)	191 (68.46)	0.0002*
Stroke (S)/TIA	37 (16.52)	54 (19.35)	0.485
Vascular Disease	224 (100)	279 (100)	1
HPL	117 (52.23)	124 (44.44)	<0.0001*
SMK	90 (40.18)	105 (37.63)	0.51
CAD	125 (55.80)	138 (49.46)	1
CABG	25 (11.16)	28 (10.03)	0.770

PCI	117 (52.23)	122 (43.72)	0.05*
Old MI	37 (16.52)	42 (15.05)	0.712
COPD	11 (4.91)	10 (3.58)	0.506
CKD	144 (64.29)	175 (62.72)	0.78
HD/PD	86 (38.39)	95 (34.05)	0.350
Cr	3.601 ± 3.282	3.002 ± 2.826	0.036*
Af	52 (23.21)	68 (24.37)	0.833
Imd	6 (2.68)	15 (5.38)	0.178
HbA1C	7.610 ± 1.939	7.097 ± 1.769	0.002*
Cholesterol	148.69 ± 39.61	150.44 ± 39.31	0.622
LDL	82.00 ± 34.18	84.46 ± 32.68	0.414
HDL	42.58 ± 14.918	43.22 ± 15.46	0.639
TG	128.89 ± 82.27	132.45 ± 85.10	0.636
Glu	152.55 ± 72.53	140.03 ± 66.56	0.046*
ASA	171 (76.34)	214 (76.70)	1
clopidgrel	194 (86.61)	233 (83.51)	0.381
cilostazol	141 (62.95)	160 (57.35)	0.234
pentoxyphilline	1 (0.46)	0 (0)	0.445

direct oral anticoagulant (DOAC)	30 (13.39)	43 (15.41)	0.610
ACEIARB	94 (41.96)	126 (45.16)	0.526
statin	121 (54.02)	162 (58.06)	0.367
Betablocker	82 (36.61)	107 (38.35)	0.711
CCB	86 (38.39)	115 (41.22)	0.523
Insulin	56 (25)	50 (17.92)	0.061
**Rutherford classification**			
1	0 (0)	0 (0)	1
2	0 (0)	0 (0)	1
3	0 (0)	0 (0)	1
4	38 (16.96)	92 (32.97)	<0.0001*
5	156 (69.64)	160 (57.35)	0.005*
6	25 (13.39)	27 (9.68)	0.659
**Target vessel**			
CIA	14 (6.25)	27 (9.68)	0,191
EIA	13 (5.28)	32 (11.47)	0.028*
CFA	11 (4.91)	16 (5.73)	0.842
SFA	131 (58.48)	154 (55.20)	0.470
ATA	117 (52.23)	131 (46.95)	0.251

Popliteal	55 (24.55)	52 (18.64)	0.125
Peroneal artery	51 (22.77)	45 (16.13)	0.067
Tibiofibular TP trunk	29 (12.95)	35 (12.54)	0.893
PTA	98 (43.75)	98 (35.12)	0.053
DPA	10 (4.46)	5 (1.79)	0.112
Plantar artery	15 (6.69)	8 (2.87)	0.053

BMI: body mass index; CHF (C): HTN: hypertension; DM: diabetes mellitus; Stroke (S)/TIA; HPL: hyperlipidemia; SMK: smoking status; CAD: coronary artery disease; CABG: Coronary Artery Bypass Graft; PCI: Percutaneous coronary intervention; MI: myocardial infarction; COPD: chronic obstructive pulmonary disease; CKD: chronic kidney disease; HD/PD: hemodialysis/peritoneal dialysis; Cr: creatinine; Af: atrial fibrillation; Imd: immune-related disease; HbA1C: hemoglobin A1C, LDL: low-density lipoprotein; HDL: high-density lipoprotein; TG: triglyceride; Glu: glucose; ASA: Acetylsalicylic acid; ACEIARB: Angiotensin-Converting Enzyme Inhibitor (ACEI)/Angiotensin Receptor Blocker (ARB); CCB: Calcium channel blockers; CIA: common iliac artery; EIA: external iliac artery; CFA: common femoral artery; SFA: superficial femoral artery; ATA: anterior tibial artery; Tibiofibular TP (tibioperoneal) trunk; PTA: posterior tibial artery; DPA: dorsalis pedis artery.

Table S4. Calibration analysis for years 1-5 based on multivariate-adjusted MCR models for MALE.

year	diff_1	diff_2	diff_3	diff_4	diff_5	diff_6	diff_7	diff_8	diff_9	diff_10	Avg.	Std. dev
1	0.034	-0.270	0.050	-0.067	0.0913	0.038	0.212	0.083	0.117	0.141	0.043	0.132
2	0.173	0.237	0.400	0.232	0.1515	0.352	0.432	0.422	0.413	0.391	0.320	0.110
3	0.074	0.425	0.487	0.475	0.3788	0.400	0.283	0.392	0.472	0.534	0.392	0.132
4	0.243	0.486	0.453	0.515	0.3101	0.431	0.429	0.498	0.539	0.479	0.438	0.093
5	0.445	0.420	0.548	0.508	0.5168	0.499	0.531	0.467	0.607	0.590	0.513	0.060

Table S5. Calibration analysis for year 1 - year 5 based on multivariate-adjusted traditional models for MALE

year	diff_1	diff_2	diff_3	diff_4	diff_5	diff_6	diff_7	diff_8	diff_9	diff_10	Avg.	Std. Dev
1	0.021	-0.282	0.062	-0.076	0.0908	0.048	0.222	0.085	0.132	0.138	0.044	0.139
2	0.146	0.253	0.387	0.209	0.1498	0.369	0.448	0.425	0.428	0.388	0.320	0.119
3	0.064	0.438	0.492	0.449	0.3946	0.416	0.307	0.387	0.478	0.518	0.394	0.131
4	0.235	0.502	0.434	0.507	0.3606	0.458	0.457	0.490	0.551	0.447	0.444	0.089
5	0.399	0.441	0.531	0.488	0.6104	0.527	0.556	0.434	0.582	0.587	0.516	0.072

Table S6. Calibration analysis for years 1-5 based on multivariate-adjusted MCR models for MACE.

year	diff_1	diff_2	diff_3	diff_4	diff_5	diff_6	diff_7	diff_8	diff_9	diff_10	Avg.	Std. dev.
1	-0.025	0.001	0.039	0.025	0.0002	-0.016	0.064	-0.092	0.072	0.072	0.014	0.052
2	0.114	0.020	0.024	0.094	0.0460	0.105	0.084	-0.010	0.021	0.022	0.052	0.043
3	0.129	-0.003	0.080	0.013	0.1947	0.127	0.130	0.049	0.095	0.093	0.091	0.059
4	0.207	0.122	0.026	0.097	0.0386	0.180	0.159	0.036	0.092	0.118	0.108	0.062
5	0.362	-0.249	0.093	0.119	0.2520	0.190	0.182	0.125	-0.098	0.189	0.117	0.174

Table S7. Calibration analysis for years 1-5 based on multivariate-adjusted traditional models for MACE.

year	diff_1	diff_2	diff_3	diff_4	diff_5	diff_6	diff_7	diff_8	diff_9	diff_10	Avg.	Std. dev.
1	-0.025	-0.001	0.037	0.023	0.0007	-0.013	0.065	-0.094	0.071	0.070	0.013	0.052
2	0.116	0.021	0.022	0.091	0.0461	0.104	0.086	-0.014	0.023	0.020	0.051	0.044
3	0.137	0.003	0.079	0.015	0.1930	0.122	0.133	0.062	0.101	0.090	0.093	0.058
4	0.211	0.121	0.024	0.108	0.0181	0.171	0.158	0.045	0.097	0.114	0.107	0.064
5	0.378	-0.249	0.075	0.111	0.2774	0.181	0.181	0.186	-0.079	0.175	0.124	0.178

Table S8. Calibration analysis for years 1-5 based on multivariate-adjusted MCR models for MALE + MACE

year	diff_1	diff_2	diff_3	diff_4	diff_5	diff_6	diff_7	diff_8	diff_9	diff_10	Avg.	Std.dev.
1	0.003	-0.253	0.060	-0.069	0.0614	0.043	0.269	0.004	0.135	0.172	0.043	0.141
2	0.272	0.319	0.381	0.276	0.1557	0.371	0.420	0.394	0.348	0.425	0.336	0.083
3	0.146	0.540	0.533	0.436	0.4538	0.524	0.416	0.351	0.453	0.477	0.433	0.116
4	0.340	0.605	0.390	0.536	0.3836	0.511	0.509	0.395	0.561	0.626	0.486	0.101
5	0.737	-0.313	0.660	0.581	0.5705	0.551	0.526	0.514	0.430	0.708	0.496	0.299

Table S9. Calibration analysis for years 1-5 based on multivariate-adjusted traditional model for MALE + MACE

year	diff_1	diff_2	diff_3	diff_4	diff_5	diff_6	diff_7	diff_8	diff_9	diff_10	Avg.	Std. dev.
1	-0.006	-0.265	0.066	-0.079	0.0603	0.054	0.274	0.006	0.149	0.170	0.043	0.147
2	0.253	0.324	0.367	0.257	0.1549	0.393	0.427	0.396	0.362	0.424	0.336	0.088
3	0.143	0.539	0.533	0.423	0.4707	0.537	0.433	0.345	0.463	0.462	0.435	0.119
4	0.341	0.605	0.373	0.536	0.4296	0.529	0.525	0.379	0.580	0.600	0.490	0.100
5	0.715	-0.296	0.592	0.568	0.6617	0.572	0.542	0.543	0.417	0.698	0.501	0.293

1. Chen, P.-S.; Lee, M.; Tang, S.-C.; Huang, P.-H.; Yeh, H.-I.; Hou, C.J.-Y.; Hsieh, I.-C.; Lee, J.-T.; Jeng, J.-S.; Li, Y.-H. 2022 focused update of the 2017 Taiwan lipid guidelines for high risk patients: Coronary artery disease, peripheral artery disease and ischemic stroke. *Journal of the Formosan Medical Association* **2022**, *121*, 1363-1370.
